# The protective and pathogenic role of Th17 cell plasticity and function in the tumor microenvironment

**DOI:** 10.3389/fimmu.2023.1192303

**Published:** 2023-06-29

**Authors:** Yuanyuan Pan, Wenjing Yang, Bo Tang, Xiaobo Wang, Qi Zhang, Weiping Li, Li Li

**Affiliations:** Department of Hematology, The Second Hospital of Dalian Medical University, Dalian, China

**Keywords:** tumor microenvironment, cancer, Th17, Treg, Th1, IL-17

## Abstract

At the turn of the century, researchers discovered a unique subtype of T helper cells that secretes IL-17 and defined it as Th17. The latest study found that Th17 cells play both positive and negative definitive roles in the regulation of antitumor immune responses. Although the function of Th17 in the tumor microenvironment remains poorly understood, more and more studies have shown that this paradoxical dual role is closely related to the plasticity of Th17 cells in recent decades. Further understanding of the characteristics of Th17 cells in the tumor microenvironment could yield novel and useful therapeutic approaches to treat cancer. In this review, we further present the high plasticity of Th17 cells and the function of Th17-producing IL-17 in tumor immunity.

## 1 Introduction

In terms of tumor immunity, CD8^+^ T cells, including CTL, directly kill cancer cells and impair tumor growth, while CD4^+^ T cells act mainly by stimulating the function of other immune cells (1–3). Naïve CD4^+^ T cells(Th0) exposed to distinctly specific cytokine surroundings have the potential ability to proliferate and effectively differentiate into a variety of epigenetic states, such as Th17, Th1, Treg, Th9, and T follicular helper cells (4–6), each with a distinct function and an individual cytokine profile. In the early 21^st^ century, Park H, Laurie E Harrington, and Annunziato et al. discovered the unique existence of an IL-17-producing CD4^+^ T cell subtype which we now term Th17 cells. This is the beginning of the research on Th17 cells (7–9). Subsequently, Th17 can be converted into other different subpopulations, mainly Th1 and Treg cells (10–12), but also into Th2 and Tfh under different conditions (13). To a large extent, Th17 cells demonstrate a considerable degree of context-dependent plasticity compared to Th1 and Th2 cells which have more phenotypic stability from the Muranski P’s research findings (11). The observed plasticity of Th17 cells is asymmetric and it occurs only in the direction of Th17 to Th1, as Th1 cells cannot easily convert to Th17 cells (11). It was previously thought that Th17 cells can play an autoimmune suppressive function by secreting IL-17 to induce angiogenesis to promote tumor growth (14). And the follow-up studies demonstrated that Th17 cells can be transdifferentiated into the Treg phenotype to exert a tumor-promoting role relying on this flexibility as well (15–17). By summarizing the studies of previous scholars, we found that this high degree of plasticity is also imperative for the antitumor activity defined for Th17 cells in the development of autoimmunity because Th17 cells can even directly convert to the Th1 phenotype and produce IFN-γ to exert anti-tumor effects. To date, there is a growing body of research indicating that Th17 cells influence the prognosis of cancer patients through their high plasticity and the secretion of inflammatory cytokines like IL-17. In this review, we briefly outline the differentiation and developmental pathways of Th17, Th1, and Treg cells, focus on our existing understanding of the implications of Th17 cell plasticity and functions, and finally summarize the specific mechanisms by which th17 functions in the tumor microenvironment.

## 2 The differentiation process of CD4^+^T cells linked to Th17 cell plasticity

CD4^+^ T cells are both essential regulators of antitumor immunity and critical protectors against invasion by external pathogens. Accumulating evidence currently elucidates the importance of the CD4^+^ T cell and their polarization status recognizing tumor antigens and resisting tumor cell proliferation in response to cancer immunotherapy (18). Luckheeram RV and colleagues put forward that, after antigen stimulation, differentiation of the naive precursors is initiated due to the interaction of TCR and CD4 as co-receptor with antigen-MHC II complexes presented by professional antigen-presenting cells (APCs) (19, 20). And continued research determined that the Naive CD4^+^T cells are activated when the TCR recognizes a specific peptide on MHC-II expressed by APCs (21, 22). The flexible differentiation options of Th0 into an individual subpopulation of helper T cells depends on cytokines secreted by APCs and other precursor cells, which induce a network of downstream signaling pathways that lead to cells’ initial activation, proliferation, and eventual differentiation of naive cells into specific effector cells. Different T cell populations that have been characterized, are tightly involved in several cancers and inflammatory diseases (23–25). In the paragraphs below we will characterize the specific differentiation process of naïve CD4^+^T cells to Th17, Th1, or Treg cells in detail.

### 2.1 Th17

As a distinct inflammatory lineage of CD4^+^ helper T cells, Th17 cells are defined by high levels of IL-17 secretion and secrete IL-17A, IL-17F, IL-21, IL-22, and CCL20 (26–29). Th17 cells are dependent on co-stimulation of CD28 and ICOS and are characterized by a master transcription regulator, the orphan nuclear receptor RORγT, which directs the transcription of the genes encoding IL-17 (27, 30, 31). Similarly, the other transcription factor, STAT3, binds to most of the relevant genes playing a crucial role in the differentiation of CD4^+^ T cells into Th17 cells (32–34).

Joshua et al. showed that individuals with Job’s syndrome (HIES) with definite mutations in STAT3 often fail to express the adequate levels of ROR*γ*T and tend to have a concomitant deficiency of Th17 cells, emphasizing the close association between STAT3 and Th17 cells (32, 35–37).

Th17 differentiation is highly dependent on the contribution of the cytokines TGF-*β*, IL-6, IL-21, and IL-1*β* (38–42), with subsequent long-term maintenance of the distinct Th17 lineage phenotype in the presence of IL-23 (43–45). In 2020, researchers find that TBK1 produced by intestinal epithelial cells blocked Th17 cell differentiation related to the regulation of IL-1*β* production, thereby preventing inflammation and tumorigenesis (46). IL-6, IL-21, and IL-23 all activate Stat3 and IRF4-dependent expression of ROR*γ*t specifically, which is crucial for Th17 cell differentiation as previously mentioned (47–52). Interestingly, TGF-*β* acts to upregulate IL-23R expression and confers responsiveness to IL-23 *in vivo* (39). Several earlier studies denied the large role of TGF-β in human Th17 cell differentiation: in a 2007 study, Sallusto stated that TGF-β even inhibited Th17 cell polarization (53). However, in 2008, experiments with serum-free cultured human neonatal CD4 T cells showed that exogenous addition of TGF-β is necessary for human Th17 cell differentiation (54).To further clarify the role of TGF-β, Annunziato conducted a further study in 2009. It was noted that TGF-β has no direct effect on the differentiation of Th17 cells from their precursors, but can indirectly promote Th17 development by inhibiting T-bet expression and Th1 amplification (55). A recent convincing study demonstrated that TGF-*β* promotes Th17 cells to differentiate by reversing SKI-SMAD4-mediated inhibition of ROR*γ*t expression through inhibition of Rorc gene acetylation (56). Furthermore, TLR2 (a pattern recognition receptor) signaling in T cells promotes Th17 cell proliferation and upregulates Th17-related genes (IL-17, IL-17F, IL-21, and CCR6) (57).

Recent studies have revealed that the heterogeneous Th17 cell subpopulation in humans can be further subdivided into two major categories based on the differential expression of chemokine receptors CCR4 and CXCR3: classical immunomodulatory Th17 and non-classical pro-inflammatory Th17. Classical Th17 is characterized by (CCR4^+^CXCR3^-^) indicating high levels of IL-17 and low levels of IFN-*γ*, whereas non-classical Th17 is characterized by (CCR4^-^CXCR3, also known as Th17.1 or Th1/Th17) producing low levels of IL-17 and high amounts of IFN -γ, with a phenotype similar to that of Th1 (13).

### 2.2 Th1

Till now, activated Th1 cells play an important role in coordinating the antitumor immune response, particularly in supporting the normalization of tumor vasculature (12, 13). Besides, Th1 cells are promoted by the critical cytokines IFN-*γ* (via Stat1) and IL-12(via Stat4), which induce the expression of the transcription factor T-bet and secretion of the signature cytokine IFN-*γ* protecting the host from intracellular infection (58).

Firstly, STAT1-dependent T-bet, a Th1-specific T box transcription factor, induces IL-12 receptors followed by activation of STAT4. It is a key regulator of Th1 differentiation and IFN-*γ* production (59–61). Secondly, Th1 cell induction is dependent on IL-12 expression, initiating downstream signaling cascades that induce IFN-*γ* production by NK cells (62, 63). Interestingly, it has been shown that cells such as Treg cells, which secrete the cytokine IL-10, regulate the life cycle of Th1 cells by preventing DCs from secreting IL-12 (64). And Interferon-gamma (IFN-*γ*), a major mediator of inflammation and tissue injury, is a fundamental product of activated Th1 cells. Although the particulars are not yet clear, there is convincing evidence provided that Th1-derived IFN-*γ* may control tumor vasculature through its anti-proliferative effect on endothelial cells and prevent tumor recurrence by keeping tumors in a state of ischemia (65). In addition, Salmonella enterica (Se) or Influenza A virus (IAV) can also influence the life cycle of Th1 cells (61).

### 2.3 Treg

Regulatory T (Treg) cells, a specialized immunosuppressive lineage, play a crucial role in the induction of tumor-specific immune tolerance (66–70). The antitumor response in B-Cell Non-Hodgkin’s Lymphoma is inhibited by intra-tumor Treg (71). It is also certain that Treg cells play a vital role in autoimmune diseases, infectious diseases, and organ transplantation (72–75).

Similar to differentiation of the Th17 lineage, the development of Treg cells is mediated by selective cytokine signals. Treg cells exert their rapid suppressive effects by secreting inhibitory cytokines such as IL-10 and TGF-*β* or by inhibitory checkpoint molecules such as TIGIT and CTLA-4 (26, 76, 77). *In vivo*, developing Treg cells also rely on the expression of IL-2, IL-15, and TGF-*β* (77, 78). In addition, in recent years, a few research has shown that Treg cells maintain immune homeostasis and support tissue function with the expression of the lineage-specifying transcription factor Foxp3 (66, 79–81). After induction of TCR stimulation, the Foxp3 shapes the identity of regulatory T cells by fine-tuning the activity of other major chromatin remodeling TFs such as TCF1 and modulates related gene expression (79, 82–85). Treg cell epigenetic alterations such as hypomethylation of specific DNA regions are also critical in the Treg cell specification process (86). Thus, therapeutic strategies implementing epigenetic regulatory drugs and gene editing technologies are expected to significantly impact Treg cells to exert antitumor effects (69, 87–89).

## 3 Role of Th17 in the tumor microenvironment

Recent studies have revealed that Th17 cells infiltrate many types of tumors, such as B cell (non-Hodgkin) cancer, breast cancer, colon cancer, gastric cancer, hepatocellular cancer, melanoma, myeloma, ovarian cancer, pancreatic cancer, and so on, depending on the chemokine receptor interactions (10, 17, 71, 90–98). As demonstrated by the studies of Meng and Yang, Th17 cells retain a high degree of plasticity, allowing for conversion to other lineages under precise stimulation or pathogenic conditions (71, 99). Because of this, we hypothesized that the high plasticity of Th17 cells may contribute to the complex dual function shaping the tumor environment.

In addition, data from Joseph Fabre confirmed the association of IL-17 with the different tumor environments (100). IL-17 is a family of pro-inflammatory cytokines produced by Th17 cells, which contains six cytokines (IL-17A to IL-17F) that are linked to five receptors (IL-17RA through IL-17RE) (14, 101–108). Activation of IL-17 and IL-17F genes by cytokine signaling appears to be functionally linked to histone H3 hyperacetylation (106). Despite the growing evidence for the pathogenic role of IL-17 in cancer, the underlying molecular and cellular mechanisms are still not fully understood. Targeting IL-17 signaling may be helpful in a variety of diseases and cancers (109–111). Here we also discuss the function of the IL-17 depending on cell or tumor type and cytokine properties in the microenvironment, focusing on the balance between the pathogenic and protective roles of IL-17 in antitumor immunity and tumorigenesis.

### 3.1 Tumor-promoting functions of Th17

#### 3.1.1 Pro-tumor functions of IL-17

Several independent studies have demonstrated that IL-17 is highly expressed in peripheral blood, malignant ascites fluid, and liver, stomach, colon, pancreas, breast, lung, and ovarian tumor tissues, and positively correlates with malignancy aggressiveness (17, 91, 94, 95, 97, 112–121). The mechanisms that we believe could achieve the oncogenic effects of IL-17 have been listed below.

Li evaluates more than 40 HCC specimens by IHC staining and finds that IL-17A levels are significantly higher in HCC specimens that have metastasized than in non-metastasized HCC primary specimens (120). That is, the high frequency of IL-17A-positive cells in tumor tissues is correlated with metastasis and poor prognosis of hepatocellular carcinoma (HCC). Further studies reveal that this correlation is since IL-17A can significantly promote cell migration rate by activating nuclear factor-κB (NF-κB) transcription factors and upregulating matrix metalloproteinase 2 (MMP2) and 9 (MMP9) (120, 122). In a recent article, Gu et al. argued that IL-17 promotes HCC invasion and migration via Akt-dependent IL-6/STAT3 activation and subsequently upregulates its downstream targets IL-8, MMP2, and VEGF (112). IL-17 enhances VEGF and CD31 expression, stimulates the transcription of angiopoietin-2, and promotes tumor growth strongly with high microvessel density (MVD) (80, 89-91). In addition, since EMT plays an important role in tumor metastasis, IL-17-induced EMT promotes lung cancer cell migration and invasion through NF-κB-mediated upregulation of ZEB1 (123, 124).

Another possible mechanism leading to cancer malignancy is that IL-17 promotes cancer stem-like cells (CSLCs) tumorigenic potential. For example, in ovarian tumors, it was found that IL-17 producing cells are located in the niche near the tumor stem cells and IL-17 promotes self-renewal of CD133(+) CSLCs mediated by NF-*κ*B and p38 MAPK signaling pathway (113). IL-17 contributes to the initiation and progression of pancreatic interepithelial neoplasia (PanIN) by increasing the activation of NF-*κ*B and MAPK and the expression of DCLK1 and ALDH1A1 (a marker of embryonic stem cells) (118). IL-17A exerts its pro-tumorigenic activity through IL-17RA which is involved in activating ERK, p38 MAPK, and NF-*κ*B signaling (115). This signals directly promotes the development of early tumor in the mice with a COX2P-NLS Cre recombinase transgene and a loxP-targeted APC allele (115, 125). In Multiple myeloma (MM), IL-17 activates the oncogenic p65 transcription factor, which directly represses the miR-192 gene by binding to the miR-192 promoter and also induced EMT and inhibits cell adhesion to fibronectin and collagen I, promoting cell migration (126). More recently, it was shown that Th17 and IL-17 also accelerate endogenously arising lung tumors proliferation and angiogenesis in part by transducing NF-*κ*B and ERK signaling to induce the expression of G-CSF, Bv8, and VEGF, among others (17). Myeloid derived suppressor cells (MDSC) as an immature form of myeloid cells mostly identified as CD11b and Gr-1 double positive cells in mice are commonly considered that can suppress immunity by arginase-1 (Arg-1), MMP9, and S100A8/A9 in tumor microenvironment (127–130). The researchers discovered that IL-17 is required for the development of MDSC and IL-17R−/− tumor bearing mice expressed lower levels of arginase-1 (Arg-1), MMP9, and S100A8/A9 (127). In IL-17R-deficient mice, MDSC cells decreased as CD8 T cells increased (127). Coincidentally, in a mouse model of lung cancer, in which an oncogenic form of K-ras (K-ras(G12D)), Seon found that Th17 cells preferentially accumulate in tumor tissue and increased Th17 associate with inflammation-promoted lung cancer by recruiting Gr-1CD11b myeloid cells, reducing cytotoxic CD8 T activity and promoting Th17 cell-mediated inflammation (17, 127).

#### 3.1.2 Plasticity of Th17 and Treg

As the constitutive ratio of Tregs and Th17 cells is modified in the tumor microenvironment, human Th17 cells exhibit substantial developmental plasticity and differentiate into Treg cells, an immunosuppressive subset infiltrating the tumor microenvironment (69, 131). The reduction of Treg cells in tumors is associated with a marked increase in survival (72).

Yang et al. found that Foxp3 expression in Treg cells obtained by *in vitro* induced differentiation was consistently increased by stimulation with TGF-*β* alone; however, in the presence of TGF-*β*, IL-6 alone or combination with IL-1 and IL-23 significantly downregulated Foxp3 expression and increased IL-17 production (132). *In vitro* experiments by Jian Ye’s group confirms this idea: first, they obtained a Th17 subpopulation by stimulating TILs from ovarian and colon cancers with a CD3 monoclonal antibody as well as IL-2; and then, they used flow cytometry to detect the results after amplifying this subpopulation three times and found a significant increase in the FOXP3^+^ cell subtype with an obvious decrease in the IL-17^+^ cells, a differentiation that is dependent on TCR stimulation (133, 134). These experiments evidenced that Th17 and Treg are capable of interconversion *in vitro*.

In biopsy specimens from B-Cell Non-Hodgkin’s lymphoma, malignant B cells induce Foxp3 expression, promote Treg development, and lead to suppression of Th17 cells differentiation, thereby establishing a profoundly inhibitory tumor microenvironment (71). This implies that there is an intimate relationship between Th17 and Treg in the tumor microenvironment.Th17 cells are a source of tumor-induced Treg cells in tumor-bearing mice (135). ID8A ovarian cancer and MC38 colorectal cancer cells were injected intraperitoneally in IL-17A^Cre^R26R^ReYFP^ fate reporter mice which allow the visualization of cells that had activated the IL-17 program irrespective of current production of this cytokine, and then researchers calculated the cells from tumor-bearing mice by flow cytometry (135, 136). The study found that a considerable proportion of eYFP^+^ cells(which represent IL17 producing cells) begin expressing Foxp3 and the percentages of Foxp3^+^CD4^+^ T cells(ex-Th17) gradually increases, while the percentage of eYFP^+^Foxp3^neg^ cells (‘true’ Th17 cells) declines at the later time points (135). In addition, the percentage of tumor-associated Foxp3 cells is significantly reduced in RORγ^neg^ID8 tumor-bearing mice (135). Stephanie Downs-Canner et al. suggested that Th17 cell transdifferentiation serves as an important pathway of Treg cell emergence in the tumor microenvironment. Recent articles have discovered that HIF-1*α* regulates the downstream Th17 genes by directly inducing ROR*γ*t transcription to promote Th17 differentiation. Eric V Dang further found that Foxp3 is very sensitive to hypoxia and HIF-1*α* inhibits Treg differentiation through the glycolytic pathway, allowing Foxp3 protein to be degraded (47, 137). There is increasing evidence that tumor-infiltrating Th17 cells can also differentiate into Tregs due to alter epigenetics and reprogram gene expression profiles, including lineage-specific transcriptional factors and cytokine genes (71, 133).

**Figure 1:**
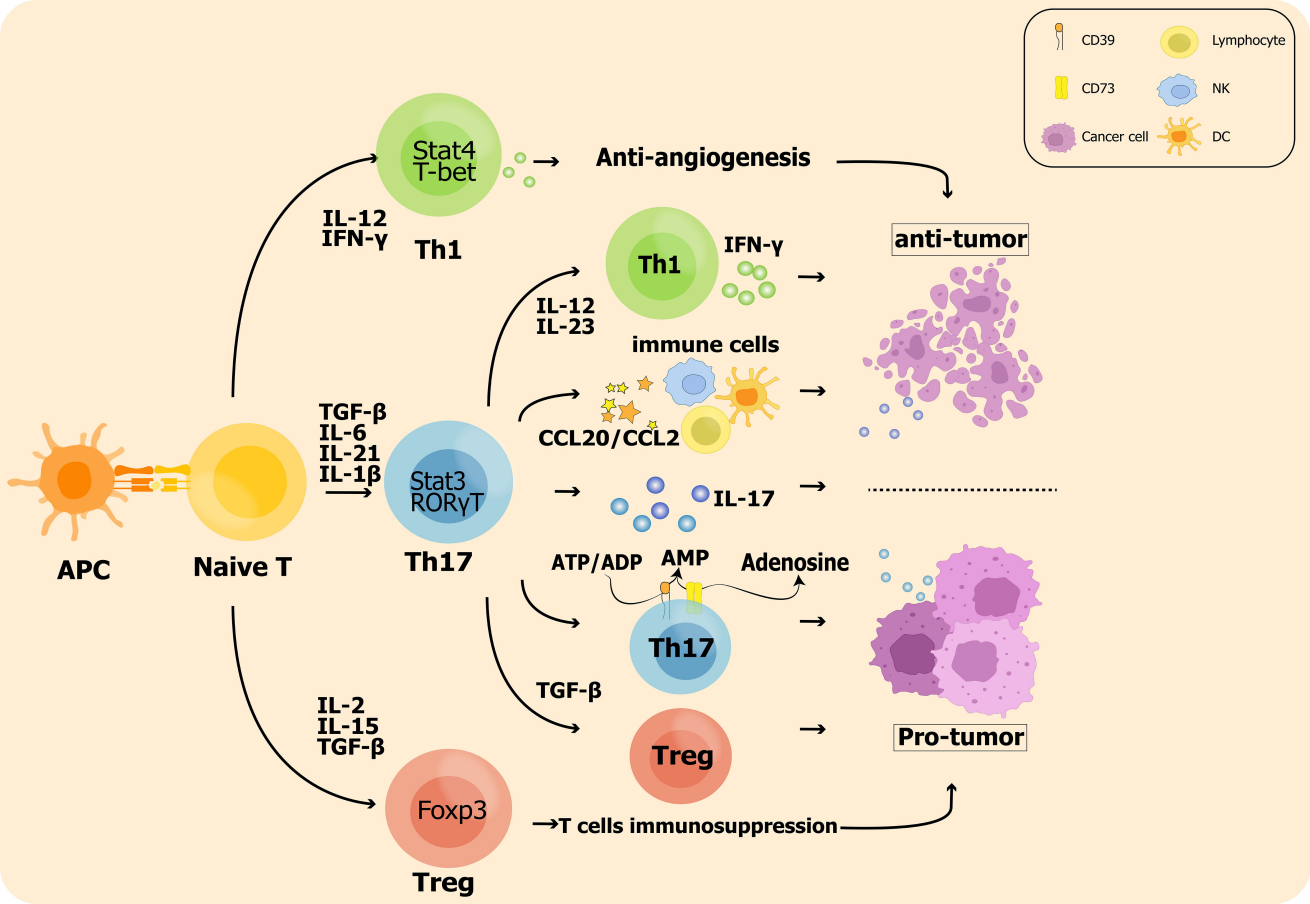
The impact of Th17 cell plasticity in the tumor microenvironment: On the one hand, Th17 cells promote Treg cell polarization leading to enhanced antitumor immunosuppression; on the other hand, Th17 cells are also converted to the Th1 phenotype and produce IFN-γ to exert antitumor effects. In addition, inflammatory hallmark cytokines such as IL-17 produced by Th17 cells are significantly associated with tumor metastasis and prognosis.

Immunosuppressive Treg cells are associated with advanced invasion and prognostic exacerbation of malignancies in tumor microenvironment as they inhibit the killing of tumor cells by antigen specific CD8^+^ T cells. Sadna Budhu et al. used ex vivo three-dimensional collagen-fibrin gel cultures of isolated B16 melanoma to qualitatively measure inhibition. Tumor cells displayed resistance to killing by activated antigen-specific CD8+ T cells, and this resistance was dependent on the binding of TGF-β above resident Treg cells in mice (138). Chen BJ et al. showed that Treg cells ultimately allow tumor escape by rendering T cells dysregulation and by their own immunosuppression (73). Therefore, shifting the balance of Th17/Treg towards Th17 may be beneficial for patients with aggressive tumors (135, 138, 139). The impact of Th17 cell plasticity in the tumor microenvironment is shown in **Figure 1**.

#### 3.1.3 CD39 and CD73 define the adenosine production of Th17

Recent studies have confirmed that Th17 cells might also make a difference in immunosuppressive functions through ectonucleotidases CD39 and CD73 to suppress T cell proliferation and cytokine production, causing pro-tumorigenic functions (140–144). The specific possible mechanism is that the enzyme CD39 cleaves ATP or ADP to AMP; meanwhile, CD73 converts AMP to adenosine, the final immunosuppressive molecule (145). Adenosine inhibits both NK and CD8+ T cells mainly by A2A adenosine receptor signaling, promoting tumor immune evasion and escape (146). Chalmin (2012) validated that *in vitro* Th17 cells generate with the cytokines IL-6 and TGF-*β* expressed CD39 and CD73, and convert ADP to adenosine, leading to suppression of CD4^+^ and CD8^+^ T cell effector functions and subsequent promotion of tumor malignancy (144). In 2014, Aiping Bai’s group presented their novel findings that dual expression of CD39 and CD161 better defines the Th17 profile of human CD4^+^ T cells, which modulates human Th17 responsiveness by altering the biological activity of acid sphingomyelinase (ASM) and activating downstream signals (including STAT3 and mTOR), as seen in patients with Crohn’s disease (147–149).

However, it was noted that CD73 and CD39 expression was downregulated and exerted anti-tumor immunity when small molecule RORt agonists were applied to induce Th17 differentiation; this experimental result seems to be contradictory with the aforementioned conclusion that Th17 promotes tumors through CD73 and CD39 (150). Th17 exerts pro-tumor effects based on CD39 and CD73 dependent on the conditions of Th17 differentiation. At high concentrations of TGF-β, transcription factor GFi-1 is down-regulated while expression of exonucleases CD73 and CD39 is increased, followed by increased adenosine production, leading to a diminished antitumor effector function (144). However, in the presence of low concentrations of TGFβ with IL-6 and IL-1β, the generated Th17 cells expressed lower levels of CD73 and better antitumor activity (151); Not coincidentally, the application of small-molecule RORt agonists resulted in decreased expression of both CD39 and CD73, th17 which in turn exerted anti-tumor immune effects, regardless of TGF-β concentration (150). In addition, this complex dual role could also be due to the alteration of the balance between th17 and treg under various culture conditions. Most human Treg cells express CD73 with CD39 and exert immunosuppressive effects, and increased Treg infiltration in the tumor microenvironment enhances adenosine-increased mediated immunosuppression. In studies applying RORyT agonists, Treg cell differentiation was inhibited which further affected CD73 & CD39 expression. Interestingly, the differentiation of Th1 cells was not inhibited in this experiment and the expression level of T-bet, the main transcription factor of th1 cells, was extremely similar to RORγT and exerted an anti-tumor immune effect.

### 3.2 Tumor-protective functions of Th17

It has been shown that the proportion of Th17 cells is decreased in the malignant tumor environment compared to the benign tumor (26, 152, 153).

As evidenced by mouse experiments *in vitro*, enhancing Th17 cell differentiation by the application of ROR*γ* agonist can increase the expression of the signature 17-type cytokines IL-17A, IL-17F, IL-22, and GM-CSF, and reduce the expression of PD-L1 (150). And then further increase the expression of co-stimulatory receptors, which may provide superior antitumor activity and represent a promising immunotherapeutic approach for the treatment of cancer (150, 154). In other words, Th17 cells affect the prognosis of patients and may even play a powerful role in anti-tumor immunity (90, 155).

#### 3.2.1 Anti-tumor functions of IL-17

While it has been reported that IL-17 promote tumor progression and metastasis by increasing inflammatory angiogenesis, other investigators have pointed out that IL-17 exert antitumor effects by modulating adaptive immune responses via recruiting T lymphocytes, enhancing NK cell activity, and promoting the generation and activation of CTLs (156–162).

**Figure 2:**
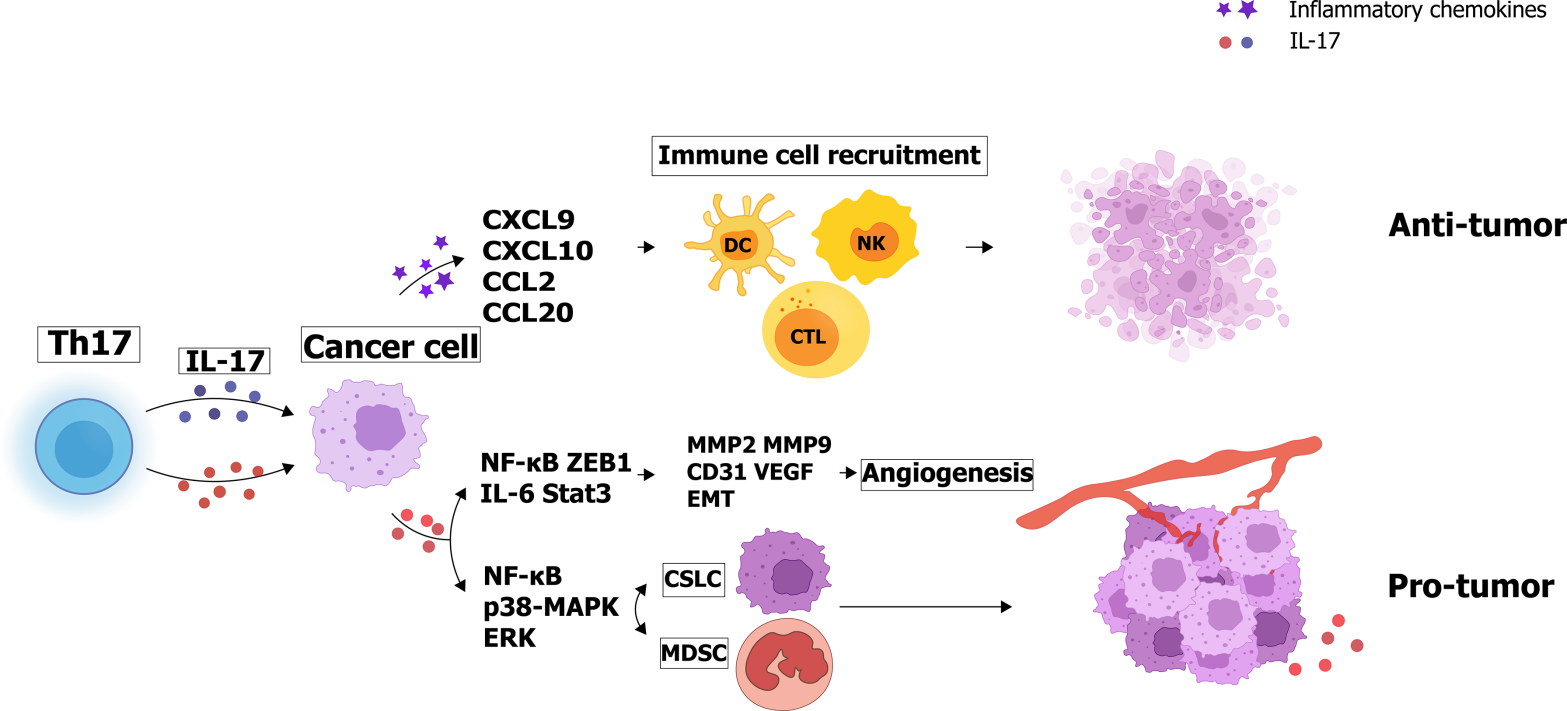
Paradoxical effects of IL-17 produced by Th17 cells: IL-17 secretes angiogenic factors and increases matrix metalloproteinase expression to promote tumor cell proliferation and invasion. Interestingly, IL-17 can also enhance tumor cell apoptosis by recruiting immune cells such as NK cells and CTL cells.

For instance, a 50-year-old man with a history of mild psoriasis and Crohn’s disease was treated with a PD-1 blocker (pembrolizumab) for metastatic colon cancer. There was a significant 50% reduction of CEA level in the first two rounds of treatment. However, after the third cycle of pembrolizumab, the patient developed a severe psoriatic rash covering 75% of the body, along with abdominal pain and increased stool frequency. To address the skin manifestations, the patient was treated with an IL-17A blocker (secukinumab) for the treatment of psoriasis (163). Although IL-17 blockade improved psoriasis symptoms and gastrointestinal pain, antitumor activity decreased as serum CEA returned to pre-treatment levels (164). In 2009, Ilona et al. inoculated mice with colon cancer MC38 cells line and found that IL-17-deficient mice had low level of tumor-infiltrating IFN-γ T cells and enhanced tumor growth and subcutaneous and pulmonary metastasis compared to normal wild-type mice (165). And Chen concluded that low amounts of intertumoral IL-17 expression may indicate a poor prognosis for patients with gastric adenocarcinoma (157). Similar results were also obtained in esophageal squamous cell carcinoma (ESCC): the density of IL-17^+^ cells was inversely correlated with tumor invasion, and the level of IL-17^+^ cells was positively correlated with the density of CD8^+^ cytotoxic T lymphocytes (CTLs), CD57^+^ natural killer (NK) cells and dendritic cells (DCs) by recent studies (156, 166, 167). The levels of IL-17^+^ TIL and other immune cells involved in antitumor effects, such as CTL, were evaluated in 181 ESCC patients using immunohistochemical methods (167). The results shows that IL-17 induces ESCC tumor cells to produce inflammatory chemokines like CXCL9, CXCL10, and CCL2, CCL20, which are respectively associated with the migration of T cells, NK cells, and DCs (167). The paradoxical effects of IL-17 produced by Th17 cells are illustrated in **Figure 2**.

#### 3.2.2 Plasticity of Th17 and Th1

Th17 cells in tumor immunity not only promote the activation of cytotoxic T cells, but also even directly convert to T helper type 1 lineage leading to the production of IFN-*γ* (10, 168). IFN-*γ* plays a key role in autoimmune diseases and tumor-protective functions through direct anti-proliferation, pro-apoptosis, and anti-angiogenesis functions (64, 169, 170).

IL-17 producing Th17 cells can switch to effectors with similar characteristics to Th1 cells (known as ex-Th17 or non-classical Th1 cells), which support the ability of antitumor or immunity (11, 170–172). New research shows that the percentage of ROR*γ*t cells is similar to that of cells expressing T-bet in tumor-infiltrating lymphocytes (TILs) from different tumor types, such as breast cancer and ovarian cancer (150). *In vitro* experiments have suggested that in the presence of low levels or total absence of TGF-*β*, IL-12 and IL-23 cytokines induce conversion of Th17 cells to the Th1 phenotype; while sufficient TGF-*β* retains Th17 phenotype is retained (12, 63). Interestingly, other researches show that by activating stat4, IL-12 and IL-23 convert Th17 precursors into the Th1-type phenotype, because of their functions of STAT4 activation (63). Furthermore, Meng revealed that Dendritic cells (DCs) expressing Notch ligand DLL4 can promote Th1 and Th17 differentiation by directly activating the transcription factors T-bet and ROR*γ* (99). Moreover, th17-derived cells (ex-Th17 or non-classical Th1 cells) distinguish from classical Th1 cells via unique surface markers, including CD161, CCR6, and IL-17RE (26, 171, 173). Basdeo SA et al. found that these non-classical Th1 cells express significantly higher anti-apoptotic Bcl-2 implying better survivability, and secrete more TNF, IL-2, GM-CSF, and IFN-*γ* (174).

#### 3.2.3 The recruitment and activation of immunity cells

Th17 cells also drive antitumor immune responses by recruiting immune cells to the tumor, particularly by activating effector CD8^+^T cells. It was reported that Th17 cells stimulated CD8^+^ cytotoxic T lymphocytes (CTL) responses via IL-2 and pMHC I and the deterioration of OVA-expressing B16 melanoma can be avoided by this pathway (168, 175). What’s more, Th17 cells were able to stimulate the expression of chemoattractants CCL2 and CCL20 in lung tumor microenvironments and promote the recruitment of various inflammatory leukocytes (DCs, CD4^+^, and CD8^+^T cells).It has been shown that Plasmacytoid dendritic cells (pDC) activated by CpG-activated and antigen presentation induced the differentiation and development of Th17 cells, producing large amounts of other inflammatory cytokines such as IFN-γ (176, 177). When pDCs are deficient in MHC II expression, the frequency of infiltrating Th17 in tumor tissue is reduced and the Th17 response is defective, leading to a decrease in the recruitment of immunity cells such as CTL, which ultimately leads to tumor growth (178).

Further understanding of the characteristics of Th17 cells in the tumor microenvironment could generate new therapeutic approaches for cancer treatment. In contrast to Th1 cells, Th17 cells do not exhibit senescence or apoptosis and maintain potent anti-tumor efficacy *in vivo* (179). Th17 cells offer promise for the next generation of ACT trials because of their stability and persistence (179).

## 4 Conclusion

It was found that Th17 cells transform into Th1 or Treg cell subtypes under certain conditions, and there are at least two balances in the microenvironment. One is the Treg/Th17 balance: the presence of TGF-*β*, IL-23, and IL-6 maintain the Th17 phenotype, while TGF-*β* alone allows a shift in differentiation toward the Treg phenotype (67). The other is the Th1/Th17 balance: IL-12 and IL-23 induce the conversion of Th17 cells to a Th1 cells phenotype in the absence of TGF-*β*. These two balances are in opposition to each other, shaping the effect of Th17 on tumors. Th17 cells may inhibit T cell proliferation and cytokine production through CD39 and CD73, or promote differentiation of Treg cells, resulting in altered immunosuppressive functions. However, Th17 cells may also promote the activation of cytotoxic T cells or convert to the Th1 phenotype and produce antitumor effects of IFN-*γ* in tumor immunity. Given the role of protumoral Treg and antitumoral Th1 on tumors, we believe that further understanding of the high plasticity of Th17 cells may yield new therapeutic approaches to treat cancer (180).

In addition, this two-fold effect also depends on IL-17. IL-17 is significantly correlated with tumor development as well as prognosis, and the relationship between prognosis and IL-17 expression level differed among tumor types (181). On the one hand, IL-17 serves to promote tumor cell proliferation or invasion and inhibit tumor cell apoptosis by secreting angiogenic factors and increasing the expression of matrix metalloproteinases (including MMP-2 and MMP-9). On the other hand, IL-17 enhances apoptosis and decreases tumor growth by promoting the activation of NK cells and CTL cells, recruiting neutrophils, NK cells, and CD8^+^ T cells (182). The effects of Th17 cells are concluded in **Table 1**. 

**Table 1:** The pro and anti-tumor effects of Th17 cells.

		Mediator	Mechanism	Reference
Pro-tumor	IL-17	Akt-dependent IL-6/STAT3	upregulates IL-8, MMP2 and VEGF	(112)
	IL-17	VEGF, CD31, angiopoietin-2	high micro-vessel density	(79, 87, 90, 91)
	IL-17	NF-κB-mediated upregulation of ZEB1	IL-17-induced EMT	(123, 124)
	IL-17	NF-κB, p38 MAPK	promotes self-renewal of CD133(+) CSLCs	(113)
	IL-17	arginase-1 (Arg-1), MMP9, and S100A8/A9	promotes the development of MDSC	(127)
	Treg	CD8^+^T cell	T cell dysregulation and autoimmune suppression	(135, 138, 139)
	Th17	CD73 CD39	inhibiting NK and CD8+ T cells	(145, 146)
Anti-tumor	IL-17	CXCL9, CXCL10, CCL2, CCL20	associated with the migration of T cells, NK cells, and DCs	(156, 166, 167)
	Th1	INF-*γ*,TNF, IL-2, GM-CSF	anti-proliferation, pro-apoptosis, and anti-angiogenesis functions	(10, 168, 174)
	Th17	IL-2 and pMHC I	stimulated CD8+ cytotoxic T lymphocytes (CTL) responses	(168, 175)
	Th17	CCL2 and CCL20	promote the recruitment of various inflammatory leukocytes (DCs, CD4+, and CD8+T cells)	(176–178)

Since the molecular mechanisms by which Th17 cells function in tumors remain unclear, we believe that further understanding of the relevant mechanisms will facilitate the exploration of novel effective therapeutic approaches and ultimately improve the prognosis of cancer patients.

## Author contributions

YP and WY contributed equally to this review. Conceptualization, LL. Investigation, YP and WY. Writing – original draft preparation, YP and WY. Writing – review and editing, BT, XW, QZ, and WL. Supervision, YP and WY. Project administration, LL. All authors contributed to the article and approved the submitted version.
